# Combined cervical transcutaneous with lumbosacral epidural stimulation improves voluntary control of stepping movements in spinal cord injured individuals

**DOI:** 10.3389/fbioe.2023.1073716

**Published:** 2023-02-02

**Authors:** Claudia A. Angeli, Yury Gerasimenko

**Affiliations:** ^1^ Bioengineering Department, J. B. Speed School of Engineering, University of Louisville, Louisville, KY, United States; ^2^ Kentucky Spinal Cord Injury Research Center, University of Louisville, Louisville, KY, United States; ^3^ Frazier Rehabilitation Institute, University of Louisville Health, Louisville, KY, United States; ^4^ Department of Physiology, University of Louisville, Louisville, KY, United States; ^5^ Pavlov Institute of Physiology, St. Petersburg, Russia

**Keywords:** spinal neuromodulation, locomotion, spinal cord injury, transcutaneous stimulation, epidural stimulation

## Abstract

**Introduction:** Lumbosacral spinal cord neuromodulation has shown the ability to restore voluntary control and stepping in individuals with chronic spinal cord injury.

**Methods:** We combined cervical transcutaneous and lumbar epidural stimulation to explore the brain-spinal connectomes and their influence in spinal excitability and interlimb coupling. Four individuals with a prior implanted lumbosacral spinal cord epidural stimulator participated in the study. We assessed lower extremity muscle activity and kinematics during intentional stepping in both non-weight bearing and weight-bearing environments.

**Results:** Our results showed an inhibition of motor evoked potentials generated by spinal cord epidural stimulation when cervical transcutaneous stimulation is applied. In contrast, when intentional stepping is performed in a non-weight bearing setting, range of motion, motor output amplitude, and coordination are improved when cervical transcutaneous and lumbar epidural stimulations are combined. Similarly, with both stimulations applied, coordination is improved and motor output variability is decreased when intentional stepping is performed on a treadmill with body weight support.

**Discussion:** Combined transcutaneous cervical and epidural lumbar stimulation demonstrated an improvement of voluntary control of stepping in individuals with chronic motor complete paralysis. The immediate functional improvement promoted by the combination of cervical and lumbar stimulation adds to the body of evidence for increasing spinal excitability and improvement of function that is possible in individuals with chronic paralysis.

## 1 Introduction

The study of epidural stimulation for restoration of motor function after severe spinal cord injury (SCI) has seen increased interest in the past decade (Harkema et al., 2011; Rejc et al., 2015; Rejc et al., 2017a; Rejc et al., 2017b; Angeli et al., 2018; Gill et al., 2018; Wagner et al., 2018; Darrow et al., 2019; Rejc et al., 2020). Similarly, transcutaneous spinal stimulation has been studied as a non-invasive method of neuromodulation both for restoration of lower and upper extremity function (Shah and Gerasimenko, 2016; Gad et al., 2017; Grishin et al., 2017; Freyvert et al., 2018; Gad et al., 2018; Gerasimenko et al., 2018; Rath et al., 2018; Sayenko et al., 2018; Benavides et al., 2020). We have demonstrated that the voluntary control of movements in persons with motor complete SCI can be recovered using epidural spinal neuromodulation (Harkema et al., 2011; Angeli et al., 2014). Following this discovery brain-spinal connectomes are beginning to receive some attention (Parhizi et al., 2021), as a potential pathway to enhance excitability of the spinal as well as cortical networks and improve interlimb coupling. Here we introduce a novel strategy that combines cervical and lumbar stimulation to neuromodulate locomotor-related spinal circuitry during weight-bearing and non-weight bearing locomotion in SCI persons. We hypothesize that combined cervical spinal cord transcutaneous stimulation (scTS) with continuous lumbosacral spinal cord epidural stimulation (scES) would enhance neuromodulation and coordination to improve function compared to each stimulation alone. In addition, it has been suggested that combined spinal neuromodulation will facilitate brain-spinal connectome, and improve voluntary control of locomotor functions.

## 2 Materials and methods

Four individuals with a clinically motor complete spinal cord injury and an already implanted lumbosacral neurostimulator were recruited for this study. The neurostimulating unit consisted of a 16-electrode array (5-6-5 Specify, Medtronic) implanted at the T11-L1 vertebral level over the lumbosacral spinal cord (L1-S1 spinal cord segments) and connected subcutaneously to the pulse generator implanted in the abdomen or flank (Harkema et al., 2011). According to the Declaration of Helsinki, individuals signed an informed consent approved by the University of Louisville’s Institutional Review Board (17.1024 MC-IS-6). All individuals were male with a mean age of 35.8 ± 2.5 years and time since injury mean of 8.7 ± 2.9 years at the time of assessments (Table 1). Individuals had received the neurostimulator on average 4.9 ± 3.61 years prior to the assessments in this study (ClinicalTrials.gov identifier NCT02339233 or NCT02307565). Two of these individuals had received prior locomotor training with task-specific epidural stimulation. Training consisted of step training on a treadmill with body weight support (1 h, 5 days per week), in addition to stand training overground (1 h, 5 days per week) (Rejc et al., 2017b; Angeli et al., 2018). All training was performed with epidural stimulation configured specifically for each task. The remaining two individuals had only received stand training and voluntary training with epidural stimulation, and no locomotor training. Stand training for the second group consisted of 2 h of standing overground (5 days per week) and voluntary movement training consisted of lower extremity movements from reclined position targeted to train motor control (Angeli et al., 2014). Participants performed all training with epidural stimulation configured specifically for each task.

**TABLE 1:** Clinical characteristics.

ID	Age (yrs)	TSI (yrs)	Implant (yrs)	Neuro level	AIS score	LT training
A45	33.6	11.7	9.7	T4	A	Yes
B23	37.6	8.9	5.5	C5	B	Yes
A101	33.7	4.7	2.4	C3	A	No
A82	38.4	9.3	1.8	C4	A	No

TSI, time since injury; Neuro level, neurological level of injury; AIS, American impairment scale; LT, locomotor.

### 2.1 Experimental procedures

**FIGURE 1:**
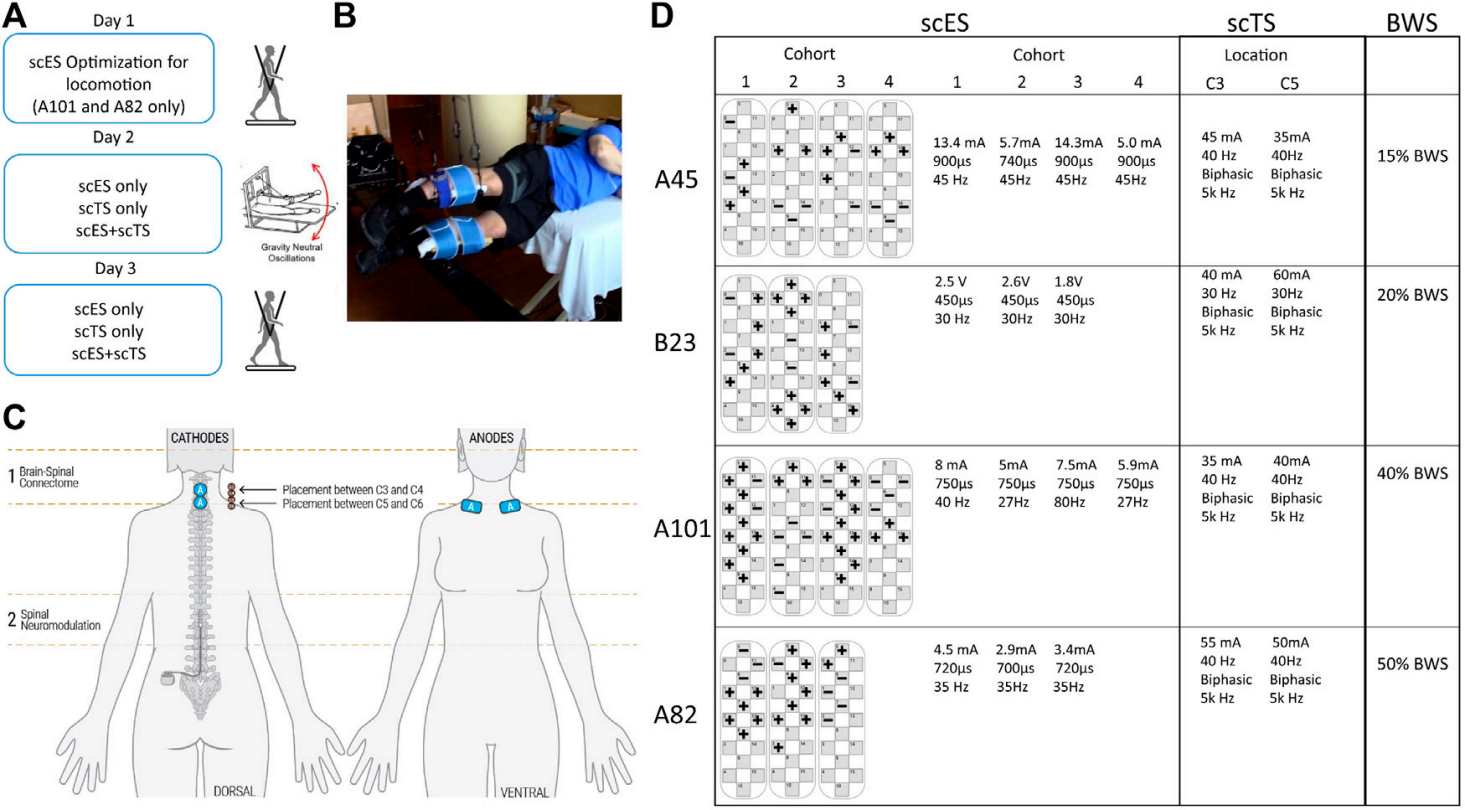
Combined neuromodulation set-up and stimulation parameters. **(A)** Timeline of assessments. **(B)** Picture of Gravity Neutral Device (GND) setup. **(C)** Graphical representation of stimulation sites showing electrode placement for scES and scTS. **(D)** Stimulation parameters for scES and scTS and body weight support (BWS) used during treadmill assessments.

We applied lumbosacral epidural stimulation (scES) with specifically configured electrode combinations through the implanted 5-6-5 Specify electrode array (Medtronic). The two individuals (A101 and A82) without locomotor training experience were first mapped for stepping using lumbosacral epidural stimulation only (Figure 1A). This consisted of stepping on a treadmill with body weight support while optimizing the scES stimulation parameters to promote independence during intentional stepping and modulate the motor output according to the step cycle.

**FIGURE 2:**
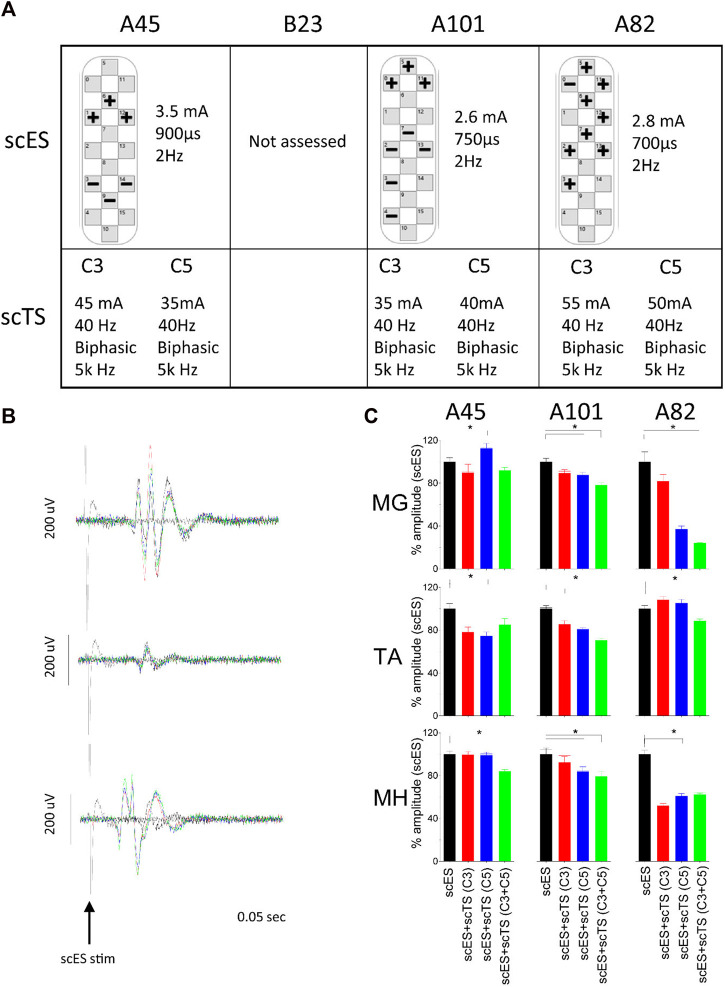
Neuromodulation of 2Hz responses. **(A)** Stimulation parameters for each participant. **(B)** Representative example of MEP for the medial gastrocnemius (MG), tibialis anterior (TA) and medial hamstrings (MH) generated by scES (black) and conditioned with scTS-C3 (red), scTS-C5 (blue) and scTS-C3+C5 (green). Peak to peak amplitude was used in data analysis. **(D)** Averaged peak–to-peak amplitude of motor evoked potentials of selected muscles during different stimulation paradigms for three of the four individuals tested. Bars represent + one Standard Error. Significant differences (*p* < 0.05) between scES alone and scES + scTS conditions are shown with an *.

All individuals were then assessed in a gravity neutral device using the step specific lumbosacral scES and cervical transcutaneous stimulation (scTS) (Figures 1C, D). The research participants were placed in a gravity neutral device while lying on their side with the upper leg supported in a sling suspended in the air directly at the shank (Figure 1B). The lower leg was placed on a free rotating brace segment attached to a horizontal board supported by vertical ropes secured to the ceiling. Transcutaneous stimulation was applied to the cervical spine (C3 and/or C5). Stimulating electrodes (2.5 cm round) were placed at midline between the spinal processes of the vertebrae. Placement was guided by palpation of anatomical landmarks such as the vertebral processes and other bony structures. Two 5 cm by 10 cm reference electrodes (anodes) were placed bilaterally on the scapula (Figure 1C). A constant current stimulator (BioStim-5, Cosyma Ltd., Moscow, Russia) was used to deliver scTS using a 1 ms train of 5 kHz (carrier frequency) biphasic square pulses repeated at a frequency of 30 or 40 Hz. scTS was applied with a carrier frequency of 5 kHz to suppress pain receptors (Ward and Robertson, 1998a; Ward and Robertson, 1998b), and minimize sensory sensitivity to scTS. Intensity for scTS was increased by 5 mA intervals up to the point of participants reported sensation on the stimulating sites and arms. scTS was mapped to enhance supra-spinal connectivity at C3-C4 and C5-C6 intervertebral spaces (Figure 1C). Research participants were asked to perform intentional step-like movements in the gravity neutral device while in the presence of scES alone, scTS alone and the combination of scES + scTS. The same conditions were also recorded during passive movement of the legs. To assess conditioning of cervical scTS on spinal evoked potentials of leg muscles scES was delivered with a single stimulation cohort configured to activate most lower leg muscles (SOL, MG, TA, MH). Stimulation was applied at 2 Hz at an amplitude close to motor threshold, while the individuals laid sideways in the gravity neutral device (Figures 1B, 2A). Each stimulation condition was recorded for 30 s.

Following all assessments in the gravity neutral device individuals were then assessed on the treadmill with body weight support using the same parameters previously identified for scES and scTS (Figures 1A, D). Participants were placed on the treadmill in an upright position and suspended using a body weight support system (PowerNeurorecovery, Louisville, KY) *via* an overhead pulley attached to a harness (Robertson, Hendersen, NV) for weight bearing stepping. Trainers provided manual assistance throughout the step cycle only when needed. A trainer positioned behind the research participant aided in pelvis and trunk stabilization, as well as appropriate weight shifting and hip rotation during the step cycle. Trainers positioned at each limb provided manual assistance by facilitating knee extension during stance and knee flexion and toe clearance during swing. Research participants were asked to perform intentional stepping while in the presence of scES alone, scTS alone and the combination of scES + scTS.

Electromyography (EMG) was used to measure motor activity from bilateral rectus femoris (RF), vastus lateralis (VL), medial hamstrings (MH), tibialis anterior (TA), medial gastrocnemius (MG), and soleus (SOL) using bipolar surface electrodes with a fixed inter-electrode distance of 2 cm. After skin preparation, electrodes were positioned parallel to the direction of muscle fibers on the belly of the muscle. Two surface electrodes placed symmetrically lateral to the electrode array incision site, over the paraspinal muscles, were used to record the lumbosacral stimulation artifact. EMG was collected at 2,000 Hz using a 24–channel hard-wired AD board and custom-written acquisition software (LabView, National Instruments, Austin, TX). Kinematics of the hip, knee, and ankle joints were acquired using a multi-camera high-speed optical motion capture system (Motion Analysis, Santa Rosa, CA) and a modified Helen-Hayes lower extremity marker model. Markers were digitized using Cortex software (Motion Analysis, Santa Rosa, CA) Ortho Trak software (Motion Analysis, Santa Rosa, CA) was used to generate 3-D joint coordinates and angles during stepping and gravity neutral assessments. During stepping assessments, ground reaction forces were acquired using a pressure sensing instrumented treadmill (Zebris, Isny, Germany) and used to identify the step cycle. Kinematics and kinetics were collected at 100 Hz sampling rate and synchronized to the EMG system.

### 2.2 Data analysis

Peak to peak amplitude of the spinal cord evoked responses was used to quantify conditioning of cervical scTS on lumbosacral spinal evoked responses generated during the 2 Hz stimulation. The middle 25–30 responses from each condition were used for analysis. Paired *t*-test of raw peak-to-peak amplitudes were performed for comparison of each scTS condition against scES alone. The linear envelope of EMG signals was computed using a lowpass filter of 10 Hz. EMG activity during stepping was quantified by the integrated signal of the EMG burst across each step cycle occurring during 30 s of the condition of interest. Similarly, burst duration was calculated as the duration of muscle activity during each step cycle. An amplitude of three times standard deviation of the mean baseline signal was used to identify onset and end of the EMG burst. Paired *t*-test were performed for comparison of scES + scTS condition against scES alone for burst duration and integrated EMG during stepping.

## 3 Results

The cervical scTS suppressed the amplitude of peak-to-peak motor evoked potentials induced by lumbosacral scES to most lower-extremity muscles (Table 2). Suppression ranging from 2%–48% of peak to peak amplitude was seen in all but three muscles for scES + scTS(C3). Similar values were observed with scES + scTS applied at C5 (6%–68% suppression). Further, the combination of C3 and C5 sites for scTS stimulation had the greatest suppression of the peak-to-peak amplitude when compared to all other stimulation conditions. All muscles showed suppression with the combination sites (C3+C5) ranging from 3% to 77% of the peak to peak amplitude. The medial hamstrings (MH) had a statistically significant suppression (<0.05) in all three participants. The amount of suppression was dependent on the muscle, as well as participant specific (Figures 2B, C).

**TABLE 2:** Paired *t*-test statistics for peak to peak amplitude during 2 Hz stimulation.

ID		SOL	MG	TA	MH
A45					
scES	Mean	217.69	225.51	84.19	650.83
	SE	8.19	8.26	4.15	19.92
scES + scTS (C3)	Mean	213.45	233.97	66.05	651.28
	SE	7.68	14.29	4.02	20.05
	t (44)	2.00			
	*p*-value	0.707	0.611	**0.003**	0.987
scES + scTS (C5)	Mean	195.64	256.81	62.89	647.99
	SE	7.50	10.89	3.16	19.72
	t (44)	2.00			
	*p*-value	0.053	**0.028**	**0.000**	0.920
scES + scTS (C3 + C5)	Mean	210.37	209.80	71.94	549.32
	SE	5.45	6.93	4.80	11.62
	t (44)	2.00			
	*p*-value	0.459	0.150	0.058	**0.000**
A101					
scES	Mean	86.33	445.34	117.87	373.06
	SE	2.78	10.66	3.45	21.47
scES + scTS (C3)	Mean	77.90	431.69	100.60	343.92
	SE	2.90	20.21	4.01	21.13
	t (42)	2.02			
	*p*-value	0.042	0.555	**0.002**	0.339
scES + scTS (C5)	Mean	76.29	418.21	94.76	312.35
	SE	2.66	19.52	2.50	16.70
	t (42)	2.02			
	*p*-value	**0.013**	**0.000**	**0.000**	**0.031**
scES + scTS (C3 + C5)	Mean	67.82	373.34	83.18	295.75
	SE	2.42	13.82	2.36	15.88
	t (42)	2.02			
	*p*-value	**0.000**	**0.00**0	**0.000**	**0.006**
A82					
scES	Mean	154.87	236.16	56.17	110.82
	SE	10.74	21.28	1.70	3.96
scES + scTS (C3)	Mean	164.06	186.98	60.98	57.28
	SE	7.10	15.01	1.68	2.46
	t (41)	2.01			
	*p*-value	0.456	0.058	**0.038**	**0.000**
scES + scTS (C5)	Mean	142.77	85.73	57.96	65.07
	SE	11.33	7.59	1.98	2.70
	t (41)	2.01			
	*p*-value	0.441	**0.000**	0.495	**0.000**
scES + scTS (C3 + C5)	Mean	98.63	54.74	50.08	68.50
	SE	6.03	0.89	1.02	1.36
	t (41)	2.01			
	*p*-value	**0.000**	**0.000**	**0.003**	**0.000**

Significant values <0.05 have been bolded.

**FIGURE 3:**
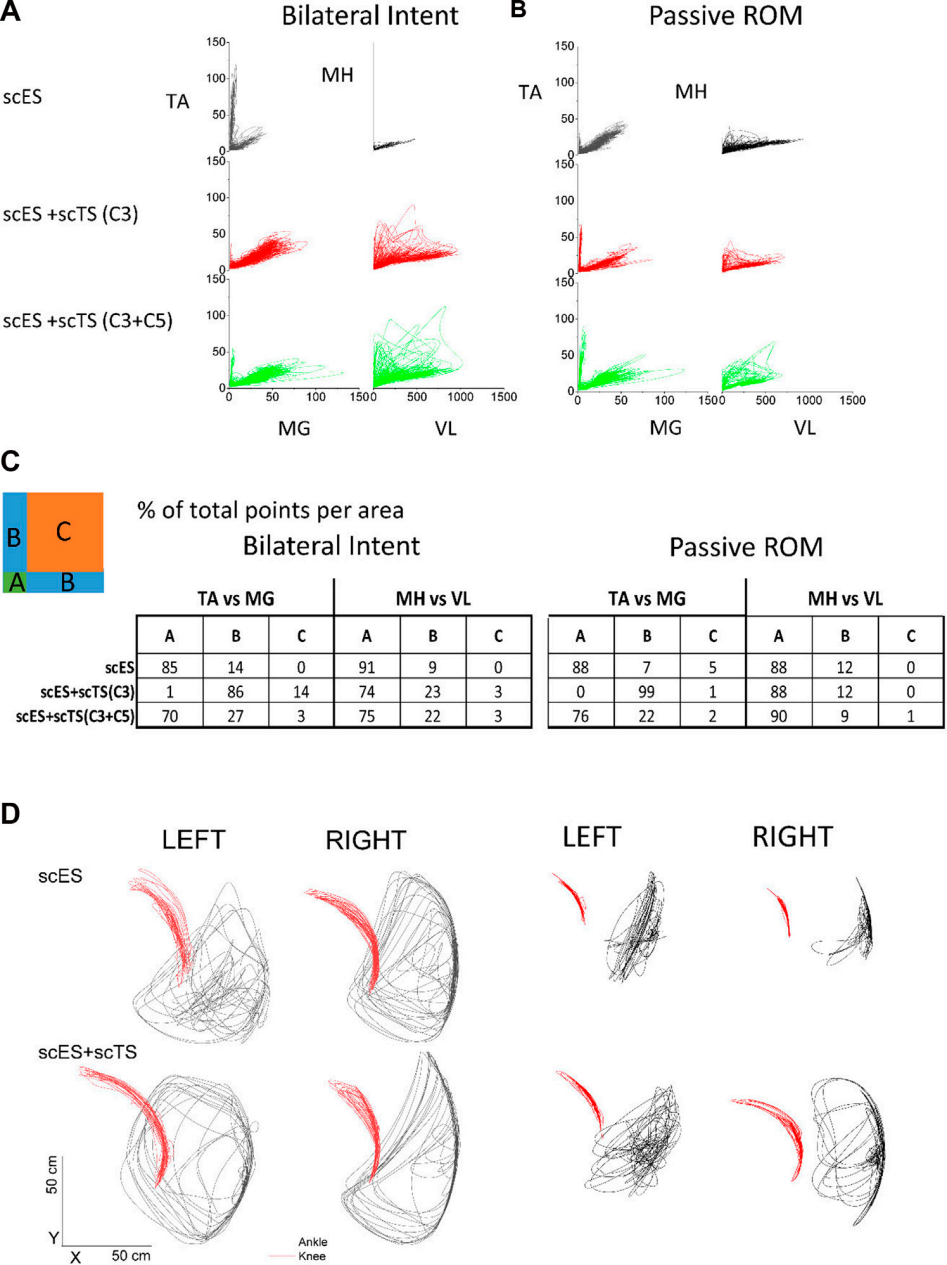
Non-weight bearing stepping neuromodulation comparison. **(A)** Flexor-Extensor coordination plot during scES only (black), scES + scTS-C3 (red) and scES + scTS = C3 + C5 (green). Graphs represent linear envelope data from top leg on the gravity neutral device during bilateral intent to perform stepping TA vs. MG (left) and MH vs. VL (right) for A82. TA, tibialis anterior; MG, medial gastrocnemius; MH, medial hamstrings; VL, vastus lateralis. **(B)** Flexor-Extensor coordination plot during same conditions as **(A)**. Graphs represent linear envelope data from top leg on the gravity neutral device during passive stepping through the full range of motion for A82. **(C)** Quantification of percent of points from linear envelope coordination plots shown in **(A)** and **(B)**. **(B)** represents the area of greatest coordination between flexors and extensors. **(D)** Sagittal plane displacement during voluntary stepping movement in gravity neutral device with scES and scES + scTS for two representative examples (B23 and A101). Ankle (black) and knee (red) joint displacement in the sagittal plane (*X*-*Y*) during scES (top panel) and scES + scTS (bottom).

Individuals were asked to perform intentional stepping in a gravity neutral device (non-weight bearing condition) in the presence of scES alone and with the combination of scES + scTS. We assessed coordination by comparing the relationship between antagonistic muscles during all attempts. In addition we assessed range of motion on the sagittal plane during all attempts. In general, the combination of scES + scTS generated a greater EMG amplitude in the extensors and improved coordination of flexors and extensors (Figures 3A, C). The medial gastrocnemius (MG) increased from a peak of 50 to 75 μV and 150 μV with scES + scTS (C3 and C3 + C5, respectively). Similarly the vastus lateralis (VL) has a peak amplitude of 500 μV with scES alone and increased to 750 and 1,000 μV with scES + scTS (C3 and C3 + C5, respectively). To assess coordination we report the percent points from the linear envelope cross-plots (TA vs. MG and MH vs. VL) in three areas. A greater number of points fell within the B areas for both of the scES + scTS conditions for both TA vs. MG (86% C3 only and 27% C3 + C5) and MH vs. VL (23% C3 only and 22% C3 + C5) muscle pairs. In one individual that had not received prior step training with scES, moving legs in passive (non-intentional) step-like movements through a full range of motion resulted in improved coordination of flexors and extensors when scES + scTS (C3 + C5) were applied (Figures 3B, C), with only changes in TA amplitude noted. During passive range of motion, coordination only improved in the TA vs. MG muscles, going from 7% of points with scES only to 99% of points with scES + scTS (C3) and 22% of points with scES + scTS (C3 + C5). Intentional step-like movement also resulted in a larger and more coordinated range of motion of the knee and ankle joint angles with the combination of scES + scTS. The left knee joint (bottom leg) has an increased displacement of 6.6, 17.96 (*X*,*Y*), and 5.7 cm, 7.53 cm (*X*,*Y*) for participants B23 and A101, respectively. The top knee had a decrease in displacement in the rostral-caudal direction for B23 -12.0 cm, 2.5 cm (*X,Y*). In contrast A101 demonstrated an increased in the X,Y displacement of the top knee of 23.5 and 27.5 cm, respectively. The displacement traces for the ankle joint show the most improvement for B23’s right leg resulting in coordinated *x,y* displacement with movement in the rostral direction from the posterior extended position similar to toe-off during walking (Figure 3D; Supplementary Video S1). The most effective scTS was the combination of two sites (C3 + C5) in all four participants tested.

**FIGURE 4:**
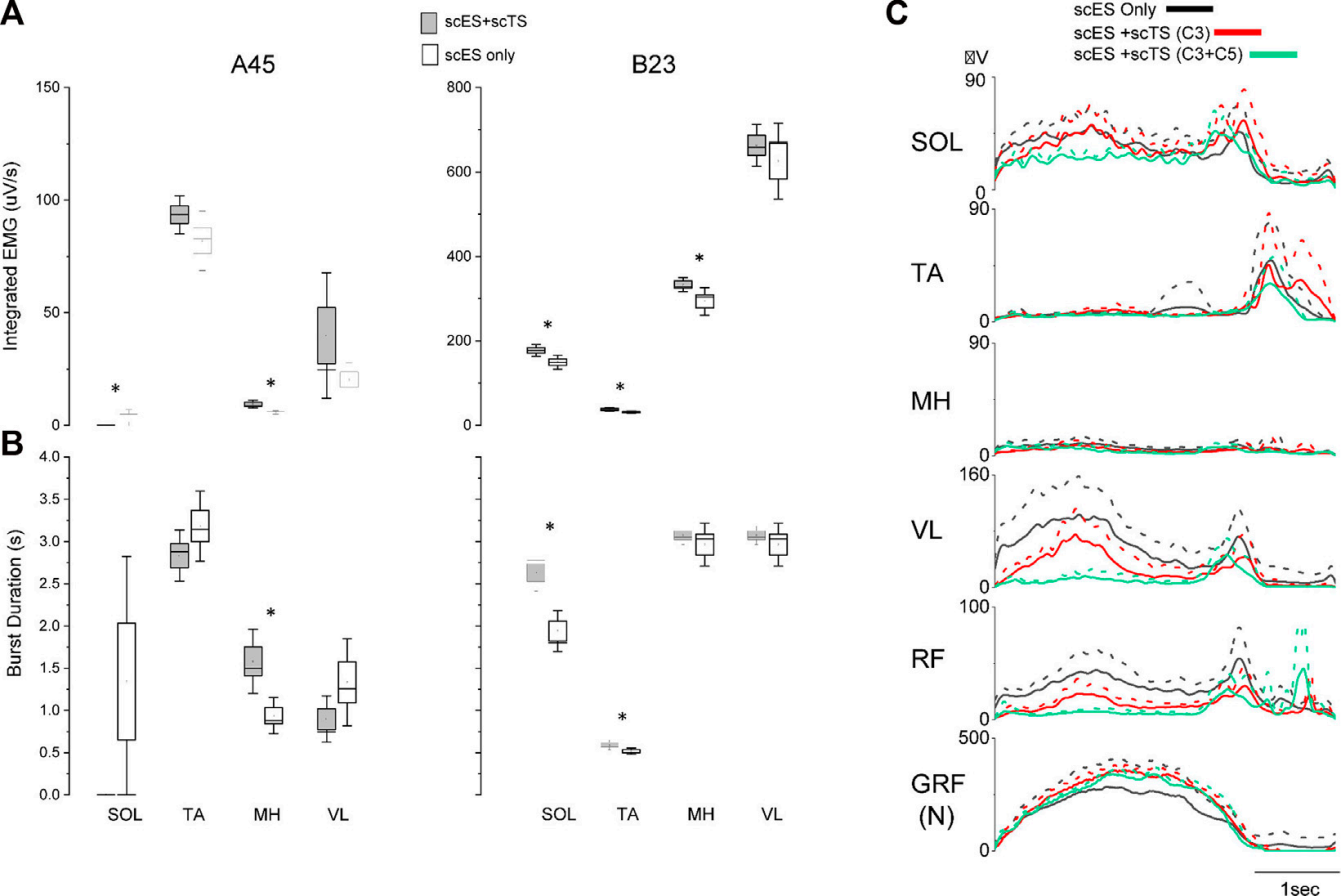
EMG changes during stepping with body weight support with scES and scES + scTS. **(A)** Integrated EMG for single leg muscles across step cycles for A45 (Left) and B23 (Right). Significant differences (*p* < 0.05) between scES alone and scES + scTS conditions are shown with an *. **(B)** EMG burst duration same for the steps represented in **(A)**. scES + scTS (gray); scES (white). Box is ± one Standard Error, line median and dot mean. Significant differences (*p* < 0.05) between scES alone and scES + scTS conditions are shown with an *. **(C)** Representative example of linear envelope of EMG normalized to the step cycle for multiple stimulation conditions in an individual without prior stepping experience. Solid line is the mean and dash line is the SD across multiple step cycles. scES only (black), scES + scTS-C3 (red), and scES + scTS-C3+C5 (green).

When individuals were assessed on the treadmill with body weight support, those that had received prior step training with scES (*n* = 2) showed the greatest improvements when the combination of scES and scTS were assessed. We observed less variability of the EMG both as demonstrated by the integrated EMG and burst duration across multiple steps when both stimulations were present (Figures 4A, B). These changes reached significance (*p* < 0.05) in the SOL, TA, and/or MH muscles. Greater coordination between left and right sides as demonstrated by the ability to take bilateral independent steps was present when the stimulation was applied at the cervical and lumbosacral spinal cord simultaneously (Supplementary Video S2). In individuals without prior step with scES experience (*n* = 2), the EMG patterns were modulated as a result of the different stimulation conditions, however, independence or coordination were not immediately improved (Figure 4C).

## 4 Discussion

There have been reports of individuals with motor complete SCI regaining the ability to walk and to improve stepping in the presence of scES (Angeli et al., 2018; Gill et al., 2018; Rowald et al., 2022). In the present study, we for the first time investigated the acute effects of combination of lumbar scES and scTS applied at one or two cervical levels on neuromodulation of the spinal locomotor-related circuitry during non-weight bearing and weight-bearing stepping. The results of this study show that scTS applied at C3 and C5 levels has an immediate neuromodulatory effect on the spinal circuitry. When examining the effects on the MEPs generated by low frequency epidural stimulation, the combination of scTS added an inhibitory effect (Figure 2). Similar results showing inhibition of the motor evoked potentials induced by epidural or transcutaneous lumbar spinal cord stimulation during attempt voluntary activation of leg muscles in the participants with motor complete SCI have been reported. It has been suggested that descending commands may increase activity of inhibitory interneuronal circuitry within spinal sensorimotor networks in individuals with SCI (Calvert et al., 2021). Similar to our results, Benavides et al. (2020) reported intracortical inhibition during paired stimulation with cervical scTS. Barss et al. (2020) also reported a suppression of the peak-to-peak amplitude of the soleus H-Reflex when cervical scTS was applied. However, the reduction in peak-to-peak amplitude is in contrast to the excitatory effect previously reported on the H-reflex of the flexor carpi radialis during cervical, lumbar and combination stimulation (Parhizi et al., 2021). When we examine the effects of scES + scTS during intentional stepping, with and without gravity, we saw an improvement in EMG amplitudes and coordination (Figure 3). For those individuals that had received prior step training with scES the improvements in coordination where more pronounced. A possible mechanism is the interlimb coupling taking place through long descending and ascending propriospinal interneurons that have been reported to influence coordination of locomotor centers (Huang and Ferris, 2009; Frigon, 2017). Our results are similar to those reported by Benavides et al. in that regardless of the inhibition exhibited on the motor evoked potentials, there was an improvement in motor performance (Benavides et al., 2020). The improvement in coordination could be the result of inhibitory effect on antagonistic muscles inducing changes in co-activation patterns. Multiple mechanisms can be responsible for the inhibition seen during 2 Hz lumbosacral scES and the enhanced voluntary control during higher frequency scES promoted by cervical scTS. The activation of cortical networks by cervical scTS facilitating cortical descending control is a plausible mechanism. However, the impact of cervical scTS on cervicolumbar coupling after motor complete SCI requires further investigation. Further understanding of how scTS optimizes the conditions for enhancement of correct descending commands while preventing erroneous commands, and how stimulation parameters may have a different modulatory effect on the spinal circuitry will lead to the design of better stimulation paradigms.

Thus, the main result of this study is the demonstration of improvement of voluntary control of stepping abilities in paraplegic persons during transcutaneous cervical and epidural lumbar stimulation. It has been suggested that transcutaneous stimulation at cervical level (C3) enabled locomotor lumbar network through activation of upper limbs central pattern generators providing interlimb integration and in combination with C5 stimulation facilitated descending dormant systems (Benavides et al., 2020). These descending drives altogether with epidural lumbar stimulation have synergistic effect on lumbar locomotor-related network controlling the stepping performance. Besides descending control, the cervical stimulation affect cortical networks activating spinal-brain-spinal loop (Benavides et al., 2020). During intentional stepping in the presence of cervical and lumbar stimulations the brain-spinal interface takes place.

The results of this study provide additional evidence on our understanding of neural connectivity and neuromodulation of the injured spinal cord. The immediate functional improvement observed during non-weight bearing and body weight supported stepping promoted by the combination of scES + scTS in individuals with chronic motor complete SCI provide the initial evidence to explore long term plasticity associated with combination stimulation. We recognize that the sample size and heterogeneity of the participants are limitations to be taken into account when generalizing the results. Further study is needed to determine the unique contributions and cumulative effects of cervical scTS and lumbosacral scES to the modulation of sensorimotor networks. Future studies should focus on examining the plasticity and recovery potential in long-term training with scES + scTS, and evaluate its potential as a rehabilitative strategy for individuals with severe SCI.

## Data Availability

The raw data supporting the conclusion of this article will be made available by the authors, without undue reservation.

## Supplementary material

SUPPLEMENTARY VIDEO S1 Participant B23 performing voluntary stepping in the gravity neutral device (GND) with scES only and with combination of scES + scTS.

SUPPLEMENTARY VIDEO S2 Participant B23 performing stepping on a treadmill with body weight support (10%) with scES only and with combination of scES + scTS.
